# Nationwide Effectiveness and Efficiency of the National Diabetes Prevention Policy Versus the Penny-per-Ounce Excise Tax Policy on Sugar-Sweetened Beverages

**DOI:** 10.7759/cureus.55866

**Published:** 2024-03-09

**Authors:** Praneeth Bandaru, Raissa Nana Sede Mbakop, Vishnu Poojitha Ronda, Suut Gokturk, Arnold N Forlemu

**Affiliations:** 1 Gastroenterology and Hepatology, The Brooklyn Hospital Center, Brooklyn, USA; 2 Internal Medicine, Piedmont Athens Regional Medical Center, Athens, USA; 3 Radiology, The Brooklyn Hospital Center, Brooklyn, USA; 4 Gastroenterology and Hepatology, University of Nebraska Medical Center, Omaha, USA

**Keywords:** united states diabetes policies, effectiveness and efficiency, nationwide, penny-per-ounce excise tax policy, national diabetes prevention program, diabetes prevention strategies

## Abstract

Diabetes has reached epidemic levels in the United States (US). This review compared two nationwide diabetes prevention policies: the National Diabetes Prevention Program (DPP) and the Penny-per-Ounce Excise (POE) tax policy on sugar-sweetened beverages (SSBs) based on their efficiency and efficacy in reducing the number of new cases of diabetes in the US. The study made a recommendation for the implementation of one or both policies based on the comparison. The national DPP focuses on screening for prediabetes in overweight/obese individuals and having positive subjects participate in a potentially insured one-year weight loss program with CDC-approved coaches. The POE tax on SSBs on the other hand is based on taxing SSBs with the objective that it will reduce new cases of diabetes due to a lower consumption of these beverages, or a switch to healthier alternatives. Studies that predicted the impact of either policy at the national level were used to compare both policies. The incremental cost-effectiveness ratio (ICER) was calculated by dividing the difference in net costs saved by the difference in net effectiveness (diabetes cases prevented per year); thereby comparing both policies in terms of costs saved for each diabetes case prevented per year. Using only nationwide US predictions, it has been estimated that the POE tax on SSB will produce the most cost savings with a greater impact on reducing new cases of diabetes if implemented; therefore, this tax should be recommended, in addition to the already implemented DPP.

## Introduction and background

Type 2 diabetes, a chronic elevation of blood glucose levels, has reached epidemic levels in the United States (US). Each year, 1.5 million people are diagnosed with diabetes; and with current trends, one out of three Americans will have diabetes by 2050 [1-3]. It is the seventh leading cause of death in the US and causes more deaths per year than AIDS and breast cancer combined [4]. The cost of diabetes is tremendously high. Estimates from the American Diabetes Association indicated the cost of diabetes to be $327 billion in 2017, with patients spending 2.3 times more than they would in the absence of diabetes [1].

The major risk factors of diabetes include prediabetes, obesity, poor diet (including sugar-sweetened beverages (SSBs) and fat-rich foods), physical inactivity, and age [5]. Prediabetes, a condition characterized by higher-than-normal glucose levels but below the diabetes threshold, increases the absolute risk of getting type 2 diabetes by three-fold to 10-fold [5,6]. Likewise, SSBs, which are beverages to which manufacturers have added sugar sweeteners, contribute significantly to excess energy intake, add little nutritional value, and result in obesity and diabetes [7]. 

Diabetes prevention strategies are complex and have been aimed at modifying the major underlying diabetes risk factors. The World Health Organization has called for multilevel, community-wide prevention strategies to address sectors contributing to food production and marketing while shaping an environment that promotes and facilitates adequate levels of physical activity [8]. Given concerns for cost and limited resources, cost-effective strategies with the greatest impact on preventing diabetes are therefore warranted.

The National Diabetes Prevention Program (DPP) and the Penny-per-Ounce Excise (POE) tax policies (a high-risk strategy and a population-based strategy, respectively) are two strategies targeting major diabetes risk factors (prediabetes, obesity, SSBs) that have recently gained much attention. They have been widely advocated for as being the most efficient and effective strategies for reducing the burden of the disease, with a huge potential to shape environments that promote long-term healthy behavioral choices [9-12].

In this review, a policy analysis was conducted in which both strategies were described and compared, and then their combined effectiveness was reported. Comparisons of the nationwide effectiveness of each strategy were limited to nationwide simulated models (discussed alongside each strategy) because the National DPP is relatively young, and its nationwide implementation is still in progress. A recent study in 2017 by the American Diabetes Association to evaluate the National DPP since its inception in 2012 was limited by the lack of data from more than half of the National DPP participants [13]. Likewise, the POE tax on SSBs has been implemented only in a few major cities in the US and it would be difficult to project the effectiveness of this policy nationwide based on the evaluation of the policy implemented in these cities.

## Review

Description of each policy option

The National DPP

**Figure 1:**
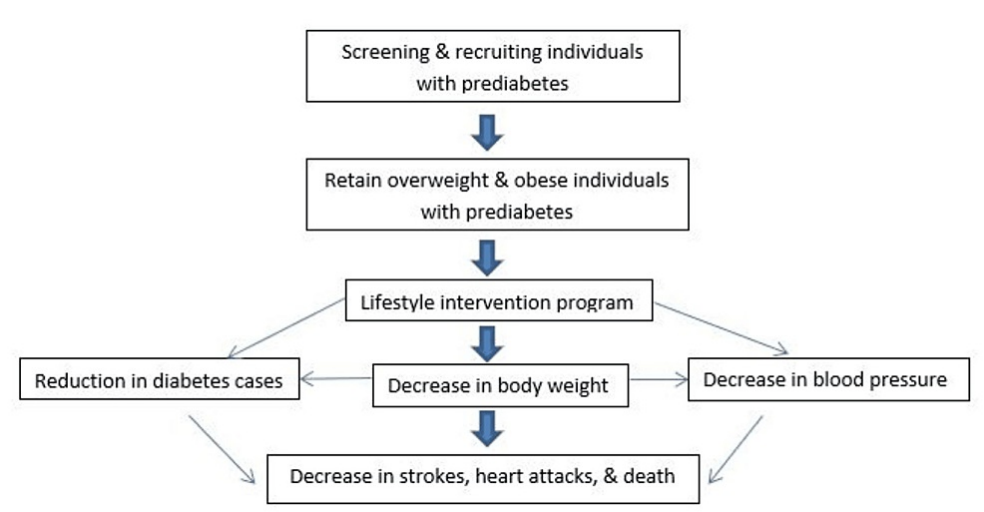
Framework for the National DPP's impact on health outcomes DPP: Diabetes Prevention Program Image Credit: Authors; Information Source: Ely et al., 2017 [13]

Background: The National DPP is the first well-organized and coordinated diabetes prevention program in the US that was passed by Congress in 2010 as the Diabetes Prevention Act of 2009 [14]. This program is a community-based lifestyle intervention program aimed at promoting weight loss reduction through dietary changes, physical activity, and behavior modification techniques in individuals identified with prediabetes with excess weight (≥ 25kg/m^2^) [15]. The goal of the program is for participants to lose 5-7% of their body weight and increase their physical activity level to at least 150 minutes per week. The program consists of an intensive series of 16 weekly training sessions where participants learn skills to reduce fatty caloric diets, increase physical activity, and learn behavior change principles. This is followed by eight monthly one-on-one or group maintenance sessions where participants are given counseling to maintain healthier choices for a maximum of 24 one-hour sessions. The intervention is delivered by trained coaches, and is designed to be implemented anywhere including virtually, office buildings, community centers, classrooms, small group gatherings, or churches. To ensure the accessibility and affordability of the program, CDC-recognized programs work in collaboration with government agencies, employers, insurers, and healthcare professionals to recruit high-risk individuals to participate in the program, while advocating for the program to be a covered health benefit by insurance companies (Figure 1) [15]

Evidence and projected impact of the National DPP: The evidence for the National DPP stems from the lifestyle intervention clinical trial conducted by the US DPP research group in 2002 [16]. They followed 3,234 overweight individuals with prediabetes for an average of 2.8 years and found that the lifestyle intervention program led to a 58% reduction in the incidence of diabetes compared to controls during follow up period. Similar findings were obtained by the Finnish national DPP program [17]. Based on these findings, several other programs replicated and adapted at a lower cost the DPP clinical trial for delivery into the community [9,18,19]. They trained lay community agents who served as DPP coaches at a lower cost and showed that every 1 kg of weight lost was associated with a decrease in diabetes incidence by 16%.

Zhuo and collaborators projected that a national community-based DPP intervention would prevent and or delay about 885,000 cases of diabetes within 25 years of implementing the program [20]. This corresponds to a diabetes incidence reduction of 7.02% (885,000/12,600,000) for individuals diagnosed with prediabetes and participating in the program (Table I). They estimated the net cost of the DPP by adding the total cost of screening for prediabetes and delivering the lifestyle intervention, and then subtracting this cost from the total cost saved by treating fewer diabetes cases due to the intervention. In their simulation model, they discounted costs and quality-adjusted life years (QALYs) for both the base case and the sensitivity analysis at 3% and 0-5% respectively. After accounting for all costs, they predicted the DPP to break even within 14 years, and to produce savings of $5.7 billion nationwide within 25 years of implementation (see Table 1).

**Table 1:** Costs and benefits of the National DPP within 25 years DPP: Diabetes Prevention Program Information Source: Zhuo et al., 2012 [20]

Benefit/cost	18-64 years	65-84 years	Total
People participating in the National DPP, millions	5.1	7.5	12.6
Diabetes cases prevented/delayed, thousands	289	596	885
Quality-adjusted life-years gained, thousands	321	341	669
Implementation cost (screening and intervention), $billion	9.1	13.1	22.3
Cost savings from averted diabetes, $billion	13.2	16.6	29.8
Overall cost savings, $billion	3.3	2.4	5.7

Issues with the National DPP: Some authors have argued that the National DPP is an individualized policy that relies much on an individual’s willingness to sustain a healthy lifestyle indefinitely after completing the program. Also, using risk scores and blood tests to identify prediabetes patients always runs the risk of excluding a good number of people who would have otherwise benefited from the DPP. However, a study by the DPP Research Group in 2015 demonstrated a reduced diabetes incidence rate by 27% during a 15-year follow-up of participants who had originally been part of the DPP trial [21], an indication of long-term benefits being sustained after the DPP trial had ended.

The POE tax policy on SSBs

Background: In 2009, the Institute of Medicine advocated for fiscal policies to help discourage the consumption of high-calorie foods with little nutritional value, including SBBs [22]. That same year, the CDC listed reducing the consumption of SSBs as one of its major strategies to prevent obesity and type 2 diabetes [23]. Taxes have been known to act on consumer behavior by influencing the different choices consumers make before purchasing a product based on its cost. Prices are expected to increase by approximately the same amount as a new tax in a competitive market [24]. An excise tax represents a tax that is placed on a particular product at the producer level and is expected to be passed on to consumers resulting in a higher shelf price of the product [25]. Given that consumers are more likely to reduce their SSB consumption if they are aware of the price increase before purchasing the product, many researchers and public health agencies have advocated for an excise tax on SSBs rather than other types of taxes such as a sales tax [26,27].

In general, economists agree that government intervention is needed when the market fails to provide the optimum amounts of goods and services for the well-being of society. This is the argument that has been applied to place taxes on alcohol and tobacco products, and there is evidence that the long-term health repercussions of consumption of food items like SSBs are similar in size and scope [10].

**Figure 2:**
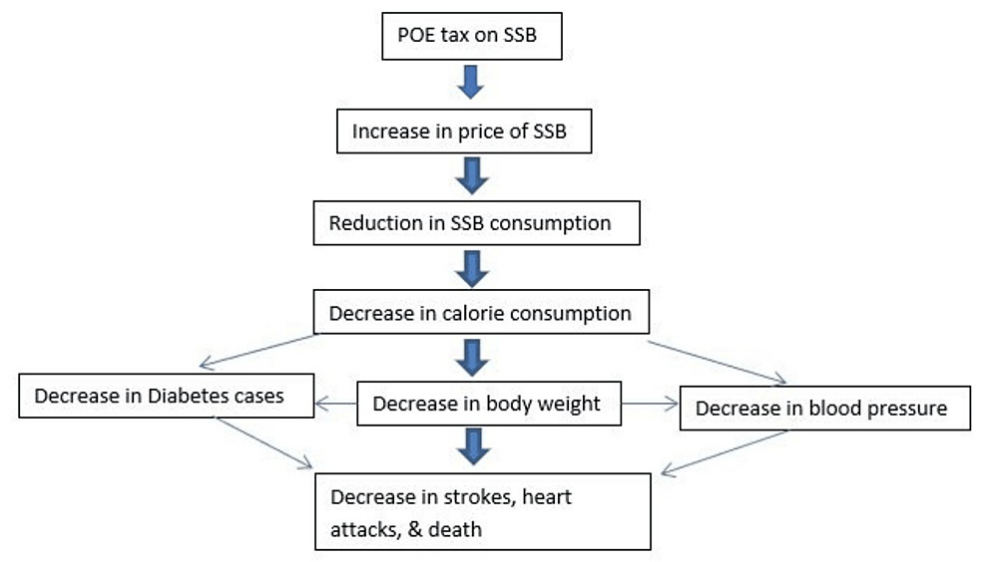
Framework for impact on POE tax on consumption of SSBs and health outcomes POE: Penny-per-Ounce Excise; SSB: sugar-sweetened beverage Image Credit: Authors; Information Source: Brownell et al., 2009 [25]

Evidence and projected impact of POE tax on SSBs: The most proposed minimum excise tax amount is the POE tax; such that a 10-ounce bottle of SSB will be levied an excise tax of 10 cents. With the current evidence that raising the cost of SSBs by 20% would decrease SSB consumption and diabetes cases [28-30], this tax is believed to be the most effective at preventing obesity and diabetes [10,25]. With an average SSB price of 4.5-5.9 cents per ounce nationwide (or $0.045-0.059), a one-cent increase per ounce (or $0.01) will correspond to a 15-25% average SSB price increase [31,32]. Figure 2 shows the impact of the tax on SSB consumption and health outcomes.

Wang and collaborators in a nationwide simulated model projected the POE tax on SSBs would reduce SSB consumption by 15% in individuals aged 25-64 years [33]. They assumed that each 10% increase in price would result in an 8% reduction in consumption of SSBs after considering that the pass-through rate of the tax to consumers may be countered by strategies such as advertising and product reformulation, and that 40% of the reductions in calorie consumption due to the tax will be offset by an increased consumption of juice or milk (foods not included in the tax). The POE tax was predicted to reduce diabetes incidence by 2.6% per year. Also, after accounting for implementation costs (producing tax statements and audits, start-up and ongoing labor costs by local, state, and federal tax administrators), they found the tax would save $17 billion in medical costs in 10 years.

Likewise in 2014, Mexico passed a modest 10% excise tax on SSBs (1 peso per liter) in a national effort to prevent diabetes and its associated burden [27]. Following its implementation, surveys demonstrated that SSB purchases declined by an average of 6% in 2014, reaching a 12% decrease by December of that year. This observed effect correlated with the soda price elasticity of Mexican consumers of -0.72 to -1.30, suggesting their 10% excise tax on SSBs would lead to a 10-13% decline in SSB consumption [27]. A study by Sanchez-Romero and collaborators projected that the 10% excise tax on soda in Mexico will prevent 189,300 new cases of type 2 diabetes among adults aged 35-94 years over a 10-year period [34], corresponding to a 4.9% reduction in diabetes incidence. They also found that the reductions in diabetes alone yielded projected cost savings of $983 million (Table 2).

**Table 2:** Costs and benefits of the excise tax for select countries Information Source: Wang et al., 2012 [33]; Colchero et al., 2015 [27], Sanchez-Romero et al., 2016 [34]

Category	United States	Mexico
Tax placed	1 cent /ounce	1 peso per liter
Estimated increase in price (pass-through rate)	10%	10%
Reduction in SSB consumption	8%	10-13%
Calorie compensation through reduction of non-taxed drinks	40%	39%
Diabetes cases prevented, cumulative incidence/year	2.6%	0.49%
Yearly cost savings, $billion	1.7	0.1

Issues with the excise tax: Concerns have been raised regarding the impact of the tax on households with low incomes. The POE tax is regressive and correlates negatively with socioeconomic status, disproportionately affecting the poor, racial, and ethnic minority groups. Moreover, this is compounded by the fact that lower-income individuals tend to consume SSBs at a higher rate compared to higher-income individuals [35], all of which may undermine the political adoption of such a policy. Therefore, to justify this tax, the tax must not only provide benefits to all but also provide increased opportunity and life chances for the poor, which is the case with this tax as the poor are also more affected by diabetes and its complications. This excise tax disproportionately provides benefits to the poor by reducing the incidence and costs of obesity and diabetes, with a reduction in outcome disparities should the tax be implemented. Thereby somewhat nullifying the regressive nature of the tax. In addition, raised income from the tax can be reinvested with a focus on low-income and minority communities, as well as fund schools and other popular projects like community centers, parks, and libraries that will benefit all. Also, based on the successful implementation of the excise tax on tobacco, there has possibly been a shift in social norms with society likely more willing to accept a similar tax on SSBs.

Comparison of both policy solutions in reducing the incidence of diabetes

Interventions for diabetes prevention or control should be prioritized as healthcare resources are limited. Both the National DPP and the POE tax policy alternatives are aimed at reducing the number of new cases of diabetes; hence it is logical to evaluate these programs based on this objective. Also, the limited resources warrant us to evaluate the efficiency of both policy solutions so as to determine the solution that provides the most cost savings whilst being efficacious at preventing new cases of diabetes. Both policy solutions were compared based on the nationwide simulation model studies described above that projected the number of diabetes cases that would be prevented, as well as their cost-effectiveness (efficiency) if either policy option was implemented at a national level.

Comparison of Program Effectiveness in Preventing Diabetes

Given that the number of diabetes cases reduced was projected at different years by the different studies, yearly projections of each study were obtained to enable comparisons between them. Also, all projections were converted to cumulative incidences to enable appropriate comparisons with a similar unit of measurement, as the comparison of counts may be erroneous.

At a national level, the study by Zhuo and colleagues projected that the National DPP will prevent 888,500 cases of diabetes within 25 years of implementation, corresponding to an incidence reduction of 7.02% (885,000/12,600,000). This represents an estimated cumulative incidence reduction of 0.28% per year (7.02÷25) [20]. On the other hand, the POE tax was projected by Wang and collaborators to reduce diabetes incidence by 2.6% per year at the national level [33]. Also, the study by Colchero and colleagues projected the POE tax in Mexico will lead to an annual cumulative incidence reduction of diabetes cases by 0.49% [27], a value greater than the projected impact of the National DPP by Zhuo and collaborators (Table 3).

**Table 3:** Predictions on national-level diabetes incidence reduction per year DPP: Diabetes Prevention Program; POE: Penny-per-Ounce Excise; SSB: sugar-sweetened beverage

Studies predicting policy impact at national levels	National DPP	POE tax on SSB
Zhuo et al., 2012 [20]	0.28%	
Wang et al., 2012 [33]		2.6%
Colchero et al., 2015 [27]		0.49%

Comparison of Program Cost-Effectiveness (Efficiency) in Preventing Diabetes

The cost-effectiveness was estimated as the cost savings per diabetes case prevented per year. This was done per year to have similar durations for comparison of both policies. In the situation where only the incidence percentage was available, the number of cases prevented was calculated using the cumulative incidence (CI) formula: CI = new diabetes cases prevented/total population at risk of getting diabetes. After these adjustments were performed, the incremental cost-effectiveness ratio (ICER) was calculated by dividing the difference in net costs saved by the difference in net effectiveness (diabetes cases prevented per year), thereby comparing both policies in terms of costs saved per diabetes cases prevented per year.

According to Zhuo and collaborators, the National DPP will prevent about 885,000 cases of diabetes within 25 years of implementing the program [20]. This corresponds to 35,400 cases prevented if considered on a yearly basis. Likewise, the program will produce savings of $5.7 billion nationwide within 25 years of implementation, also corresponding to an estimate of $228 million of cost savings per year. Also, according to the predictions made by Wang et al., the POE tax will prevent 2.6% of new diabetes cases, corresponding to 4,388,800 cases prevented per year (0.026 x 168,800,000) [33]. The intervention will yield cost savings of $17 billion over a 10-year period; an average of $1.7 billion of annual cost savings. Using predictions from both studies, the annual ICER was estimated as:

(ICER/year = annual cost saved (policy option 2-policy option 1)/annual cases prevented (policy option 2-policy option 1) 

With this formula, the ICER was estimated at $338.13 dollars saved for each case of diabetes prevented by the POE tax compared to the National DPP ((1.7 billion-228 million)/(4,388,800-35,400)). In order words, the POE tax policy will save $338.13 per year for every new case of diabetes prevented as compared to the National DPP policy solution.

Combined Effectiveness and Efficiency of Both Policies

The WHO has called for multilevel action policies for the prevention of non-communicable diseases including diabetes [8]. This is no surprise as these conditions tend to be multifactorial and develop over time necessitating combination strategies that focus on several of these factors while shaping the environment toward adopting healthier life choices. As the National DPP is almost already implemented nationwide, additional implementation of the POE tax on SSBs will prevent more than four million new cases of diabetes per year, with an incidence reduction of 2.88%. Likewise, both policies would generate cost savings of more than $1.9 billion per year if the POE is implemented.

Limitations

One limitation of the review is the fact that studies included in this paper used predictions based on simulation scenarios rather than observational data to come up with their results. Therefore, more evidence based on concrete observations is needed to better appreciate the effects each policy would have on preventing diabetes. Also, only a few studies were found that made projections for the impact of these policies at the national level. Having more studies for the comparison would have been more representative.

Also, the different studies predicting the number of diabetes cases prevented as well as the cost savings made used different age ranges to come up with these estimates. However, the studies included in this paper covered a broad range of the population that is the most at risk of getting diabetes. Also, the greater the population included in the simulation models, the higher the chances of getting more cases of diabetes prevented by the DPPs.

A third limitation is the fact that the studies used in the prediction comparisons in this paper did not provide information on the total number of people considered at risk in their simulation models. Therefore, the total population numbers used in this paper were obtained using data collected from National Health and Nutrition Examination Survey (NHANES) 2003-2006 for the National DPP study and the POE tax policies, as reported by the studies that made these predictions. It would have been more accurate if these studies had provided us directly with these numbers. However, in this paper, any ambiguous numbers obtained were not included in the comparison analysis.

Recommendation

Based on the above comparisons, there is evidence that the POE tax policy option is more effective at reducing the number of new cases of diabetes both at the state level and at the national level overall. Also, the POE policy option seems to be the more efficient of the two policies, producing more annual cost savings per diabetes case prevented. We would therefore recommend the POE tax as the better policy option of the two to be implemented or the POE tax to be added to the already existing DPP program for compounded benefits, as this policy solution is likely to have greater efficiency and effectiveness at reducing the yearly number of new cases of diabetes in the US.

## Conclusions

Given the current and projected burden of diabetes in the US, effective strategies that can be implemented at a large scale are required to prevent or control the diabetes epidemic. The most effective policy solutions are more likely to be those that target the major and modifiable risk factors of diabetes including prediabetes, overweight and obesity, SSBs, and physical inactivity. However, investing in chronic disease prevention programs requires adopting a large and long-term investment budget because many years may be required for the downstream cost savings to fully offset the up-front intervention cost. Given the current limitations in resources, policy options that are the most cost-effective at preventing as many new cases of diabetes as possible while saving the most resources are warranted. Although both solutions discussed in this paper are effective and efficient at reducing the incidence of diabetes, the POE tax policy solution seems to be the superior option at the national level. It is logical therefore to recommend the implementation of the POE tax option in addition to the already existent National DPP because not only is the POE a more viable option but also the combined effect of both policies will produce a greater impact on reducing or delaying the yearly incidence of diabetes.
